# Poetry of Medicine

**Published:** 1858-09

**Authors:** J. S. Slaughter

**Affiliations:** Author of Madeline &c., Atlanta, Geo.


### ARTICLE V.

*Poetry of Medicine.* By J. S. SLAUGHTER, Author of Madeline, &c., Atlanta, Ga.,

Poetry has thrown its brilliant halo over nearly all the achievements of the arts of peace and war; feats of personal prowess, wondrous physical endurance, mid the cold regions of the Northern lakes, or the burning tropics; the battle field with its terrible war-cloud rolling away like great volumes of smoke from the smothered furnace of gloomy Tartarus, has been visited by the muse, and the heroism of the dying, and the daring of the living are reflected in its resplendent mirror. With what thrilling interest we are carried away to the depopulated cities of Rome and Greece. We stalk along the Nile; we stand by the Euphrates, muse over the ruins of Babylon; fly on swift Pegassus, bridled at the foot of Parnassus, to the silent shores and solemn groves of Troy, while the glorious images of the muse fill us with dreamy visions, and the mouldering ruins, the massive heaps, the fallen pyramids, the path of mighty armies, the grand old mountains and hillocks, the sky itself seems whispering, breathing, glowing with poetic interest. At Rome we can almost hear the screaming of those eagles that so often perched upon her victorious banners, hear the roaring of the lions around the capitol, on the night before the assassination of Cæsar. At Troy the beautiful Helen is before us, and we commune with Ajax and Ulysses, and in the quiet groves we hear the supplications of a bereaved father to the "God of the silver bow." Nor does the interest stop here; for we are told by the poet that—

> "Brave men were living before Agamemnon."

Martyrs at the stake; brave watchmen upon the walls of Zion; the zealous missionary who carries the banner of King Emanuel to the deserts and highway places of heathen lands; these rise up before us as mighty heroes, against whom the legions of darkness and the "gates of hell itself, shall not prevail;" men who chase the fiends of darkness and revolters against the King of Heaven and the crystaline battlements.

But men have seen little in the privations, hardships, the genuine bravery, the moral heroism of the true Physician to excite interest. They see him not where "the pestilence walketh in darkness," when the great heart of humanity nerves his arm, go forth unshielded when death spreads its black wings over great cities; they see him not face the contagion, that is more formidable than a giant or a lion.

With steady nerve, a wan face, emaciated and care worn, with "red, hot eye-balls," that have had no relief in sleep, that have been exposed to gas-light, and rush-light and candle-light for nights and nights, the brave physician passes from the splendid mansion where damask curtains glowed before him, and splendid Ottomans and Brussels yielded to him, where servants as it were like black spirits, come and go at the nod; from the splendid mansion of the rich he emerges and wends his way to the little hovel of poverty, of want, and he bends over the rickety couch, and gazes earnestly in the pallid face, and touches the beating pulse, and perchance a dark shadow passes over his face, for he sees that the poor sufferer's life will soon end; or it may be, hope springs up, for he sees that the disease is giving way, what words of consolation he can utter; what tender sentiments linger upon his lips; what a champion he can be for the cause of mercy and christianity.

But what of his own peace and happiness. He enters his own domicil worn down with toil, and if the caress and smile of affection meet him, it must be abstractedly or vacantly returned, for his thoughts are with the sick; with that promising youth, who is delirious with fever, or the noble matron whose self-sacrificing disposition on behalf of those bright cherub children has sown the seeds of consumption, and who is now lingering, as it were, between life and death.

And is there not a heroism, a deep pathetic *poetry* in the practice of medicine? What genuine heroism! uncheered by martial music.

